# High-flow nasal cannula in the treatment of acute hypoxemic respiratory failure in a pregnant patient: case report

**DOI:** 10.5935/0103-507X.20180072

**Published:** 2018

**Authors:** Gustavo A. Plotnikow, Daniela Vasquez, Romina Pratto, Lucia Carreras

**Affiliations:** 1 Unidad de Terapia Intensiva, Sanatorio Anchorena - Buenos Aires, Argentina.

**Keywords:** High flow nasal cannula, Oxygen inhalation therapy, Acute Respiratory Failure, Pregnancy, Intensive care unit

## Abstract

Little evidence exists to support the use of noninvasive mechanical ventilation for acute hypoxemic respiratory failure. However, considering the complications associated with endotracheal intubation, we attempted to implement noninvasive mechanical ventilation in a 24-year-old patient who was 32 weeks pregnant and was admitted to the intensive care unit with acute hypoxemic respiratory failure and sepsis secondary to a urinary tract infection. Lack of tolerance to noninvasive mechanical ventilation led us to use an alternative method to avoid endotracheal intubation. The use of high-flow nasal cannula allowed to overcome this situation, wich supports this technique as a treatment option for critical obstetric patients that is safe for both the mother and fetus.

## INTRODUCTION

Although conventional therapeutic use of oxygen (O_2_) has long been the treatment of choice for patients with acute respiratory failure (ARF), it does not reduce respiratory work or improve alveolar ventilation and, at most, manages to deliver a fraction of inspired oxygen (FiO_2_) of less than 70%. Additionally, its dries and injures the mucosa.^(1)^

Noninvasive mechanical ventilation (NIMV) is the main treatment alternative to conventional O_2_ therapy in patients with ARF.^(2)^ In this scenario, the gas supplied to the patient can be heated and humidified, with an FiO_2_ close to 100% (in the absence of leaks). In addition, the positive pressure is able to improve gas exchange and reduce the inspiratory effort of the patient. However, it is sometimes difficult to achieve good tolerance to NIMV due to leaks around the mask, which favor the development of asynchronies between the patient and the ventilator.^(3)^ Lastly, NIMV is associated with some deleterious effects, such as delayed intubation due to masking of signs of respiratory failure.^(3)^

High-flow nasal cannula (HFNC) is a newer O_2_ therapy technique that allows the delivery of high concentrations of O_2_ and has been shown to have a positive clinical impact on patients with acute hypoxemic respiratory failure (AHRF).^(4)^ Although it does not directly apply pressure to the airways, the high flow generated by this device favors the development of low positive end-expiratory pressure (PEEP) levels and provides a continuous wash-out of the respiratory tract dead space. This effect can improve gas exchange and reduce respiratory rate (RR) and patient effort without increasing the risk of barotrauma.^(5)^ This oxygenation strategy seems particularly comfortable for the patient because the nasal cannula supplies warm and humidified gas, similar to physiological conditions, while allowing the patient to continue feeding orally and talking. However, it is still unknown whether the use of HFNC could be beneficial as a treatment strategy for AHRF in obstetric patients.

We report the case of a pregnant patient admitted to the intensive care unit (ICU) for sepsis secondary to urinary tract infection who developed AHRF and received support with an HFNC.

## CLINICAL CASE

**Figure 1:**
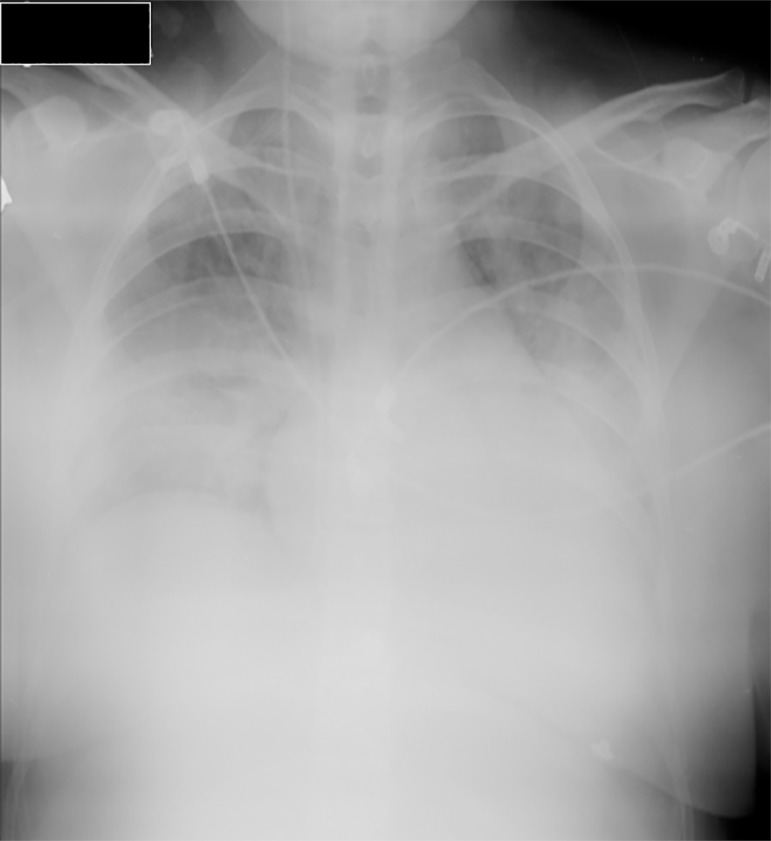
Chest X-ray (frontal view) on admission. Note the bilateral volume loss and the infiltrate on the right base.

**Figure 2:**
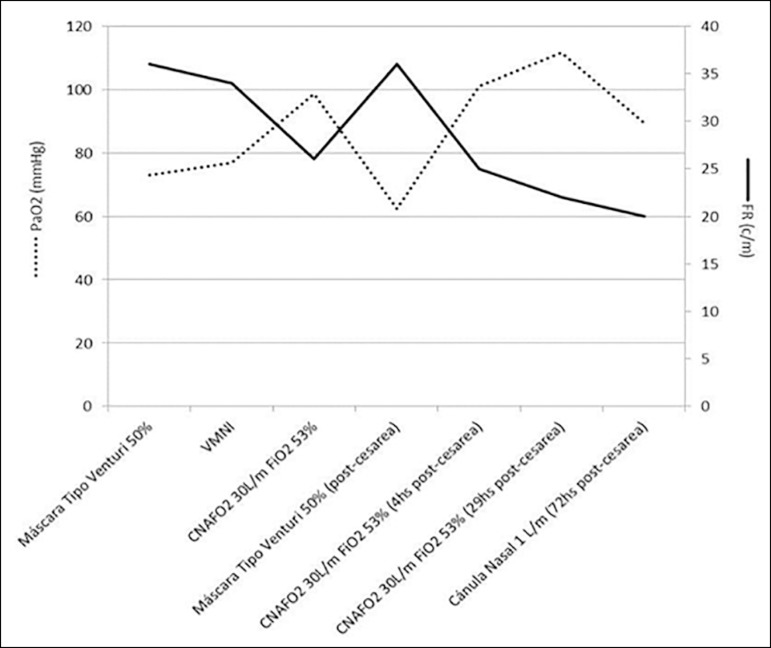
Partial pressure of oxygen and respiratory rate over time for the different devices implemented.

A 24-year-old patient at 32 weeks of gestation in her second pregnancy, with a history of recurrent urinary tract infections during pregnancy, was admitted to the ICU for sepsis secondary to a urinary tract infection with a Simplified Acute Physiology Score II (SAPS II) of 16 and an Acute Physiology and Chronic Health Evaluation II (APACHE II) score of 14. At admission, uterine contractions were confirmed. The patient reported functional class IV (FC IV) dyspnea, while arterial oxygen saturation (SaO_2_) was 92%. She was breathing spontaneously with a Venturi-type O_2_ mask at 50%, she was using accessory muscles (supraclavicular retraction), and exhibited RR of 36 cycles per minute (c/m) and heart rate (HR) of 134 beats per minute (bpm). A frontal view chest X-ray showed bilateral infiltrates (Figure 1). The condition was interpreted as AHRF in the context of sepsis due to urinary tract infection. NIMV was started, but the patient showed low tolerance to the method and to different interfaces, leading us to implement an alternative method. HFNC (AIRVO 2^®^, Fisher & Paykel, New Zealand) therapy was used initially with an inspiratory flow of 50L/minute (L/m), temperature (Tº) of 37ºC, and FiO_2_ of 100%, as indicated by the institution's protocol. The parameters were immediately adjusted according to patient's tolerance, lowering support to: inspiratory flow of 30L/m, Tº of 31ºC, and FiO_2_ of 53%. With these parameters, a significant clinical improvement was observed as evidenced by the patient's ventilatory mechanics, arterial oxygenation, SaO_2_ (97%), HR (126bpm) and especially the RR (26c/m) (Figure 2). Four hours after the start of HFNC therapy, delivery was decided due to persistence of uterine contractions and sepsis. In the operating room, the patient underwent a cesarean section with spinal anesthesia, without requiring endotracheal intubation (ETI) and using HFNC during the procedure. The neonate weighed 2,190 grams, and the 1- and 5-minute Apgar scores after birth were 8 and 9, respectively.

PaO_2_ - partial pressure of arterial oxygen; RR - respiratory rate; NIMV - noninvasive mechanical ventilation; HFNC - high-flow nasal cannula; FiO_2_ - fraction of inspired oxygen.

In the postoperative period, we attempted to discontinue HFNC therapy, but the patient quickly developed a rapid and shallow breathing pattern with the use of accessory muscles, and thus, HFNC use was reinstated, with immediate clinical improvement. Urinary tract tomography revealed a right ureteral stone. A double-J catheter was placed, and endoscopic lithotripsy was performed. Twenty nine hours after HFNC therapy was started, this support was discontinued, and O_2_ via nasal cannula at low flow was placed, with good tolerance. Three days after admission to the ICU, the patient was moved to general ward. Mother and baby were discharged home 8 and 15 days after hospital admission.

## DISCUSSION

Although the presence of sepsis during pregnancy can be considered a rare event, urinary infection is one of the main causes of nonobstetric sepsis. According to the World Health Organization, sepsis is one of the four leading causes of pregnancy-related mortality worldwide, along with hemorrhage, hypertensive disease, and abortion. Complications of sepsis during pregnancy range from premature birth, fetal infection, hypoxia and acidosis, and increased fetal mortality to a higher probability of cesarean delivery.^(6)^

In obstetric patients with ARF, evidence for the use of NIMV is not as robust^(7)^ as for other entities where it is considered the first line of treatment.^(8)^ Notwithstanding, considering the complications associated with ETI, NIMV using different interfaces was attempted- since the technical differences between interfaces could cause a lack of adherence.^(9)^ None of the options improved the patient's tolerance to NIMV.

The persistence of ARF made us to consider an alternative method to avoid EIT. The use of HFNC generates low levels of positive pressure,^(10)^ which at some point could recruit collapsed air spaces, thus decreasing the elastic load of respiratory system, improving oxygenation and decreasing muscle work associated with ventilation.^(11)^ This likely mitigated the impact of the decrease in functional residual capacity generated by the elevation of the diaphragm due to the gravid uterus in a 32-week pregnant patient, and thus optimized oxygenation.^(12)^

The decrease in RR could be associated with an improvement in alveolar ventilation and a decrease in CO_2_ concentration in conducting airways.^(13)^ Another explanation for the decrease in the RR resulting from the use of HFNC could be the generation of some type of inspiratory support,^(10)^^)^ which could result in an increase in inhaled volume and consequently a better relationship between minute ventilation and alveolar ventilation, with a resulting decrease in the RR. Although delivery and its positive impact on respiratory system,^(14)^ could have reduced ventilatory demand, we were not able to state this in our case because requirement of HFNC persisted after delivery.

The use of HFNC has been shown to improve treatment adherence, possibly because it is more comfortable. Roca et al.^(15)^ conducted a crossover study of 20 patients with AHRF and reported that the use of an HFNC was associated with greater comfort, less mucosal dryness, and lower dyspnea score than conventional O_2_ mask with a bubble humidifier. This effect in our case could have resulted in better tolerance to this method of ventilatory support. In turn, the delivery of heated and humidified gas would not only improve patient comfort but also could help in some way to decrease the metabolic cost needed to heat and humidify the inspired gas. This reduction, although impossible to quantify in the patient, could have an extremely beneficial effect.

Patients with AHRF often have high inspiratory flow rates that substantially exceed the capacity of standard O_2_ delivery systems. The ambient air carried in each inspiration dilutes the supplemental O_2_, substantially reducing the FiO_2_ delivered. HFNC generates a higher flow rate than other O_2_ delivery systems, exceeding even the peak inspiratory flow rate of the patient. As a consequence, a smaller (or negligible) mixture with ambient air is generated, allowing a more stable FiO_2_ delivery.^(16)^ This technical detail could be decisive, especially in obstetric patients with AHRF, where the minimum oxygenation target should be higher than that typically tolerated (partial pressure of arterial oxygen ≥ 70mmHg and SaO_2_ ≥ 95%) to ensure adequate O_2_ delivery to the fetus.

As with any intervention, the implementation of this technique has risks. Although in this case the use of HFNC was successful, retrospective observational studies have shown that in patients with AHRF who fail, delayed ETI is associated with poorer outcomes.^(17)^ Moreover, obstetric patients have a decreased gastric emptying rate, increased intra-abdominal pressure, and decreased upper esophageal sphincter tone, which are all variables that increase the risk of aspiration; thus, it is extremely important to maintain the patient in a semisitting position during the use of HFNC and avoid, as much as possible, the recumbent position.^(18)^

## CONCLUSION

This case report describes the successful use of high-flow nasal cannula therapy for respiratory treatment of a pregnant patient with non-obstetric sepsis, where both mother and child achieved good outcomes. We proved that in an appropriate scenario, with strict control, the implementation of high-flow nasal cannula therapy in critically ill obstetric patients with acute hypoxemic respiratory failure may be an alternative treatment option to noninvasive mechanical ventilation that has a strong foundation. High-flow nasal cannula is a tool capable of preventing endotracheal intubation in these patients while maintaining adequate levels of oxygenation.
